# Defective Peripheral Nerve Development Is Linked to Abnormal Architecture and Metabolic Activity of Adipose Tissue in Nscl-2 Mutant Mice

**DOI:** 10.1371/journal.pone.0005516

**Published:** 2009-05-13

**Authors:** Karen Ruschke, Henning Ebelt, Nora Klöting, Thomas Boettger, Kay Raum, Matthias Blüher, Thomas Braun

**Affiliations:** 1 Institute of Physiological Chemistry, University of Halle-Wittenberg, Halle, Germany; 2 Department of Medicine, University of Leipzig, Leipzig, Germany; 3 Julius Wolff Institute and Center for Musculoskeletal Surgery, Charité-Universitätsmedizin Berlin, Berlin, Germany; 4 Max-Planck-Institute for Heart and Lung Research, Bad Nauheim, Germany; University of Parma, Italy

## Abstract

**Background:**

In mammals the interplay between the peripheral nervous system (PNS) and adipose tissue is widely unexplored. We have employed mice, which develop an adult onset of obesity due to the lack the neuronal specific transcription factor Nscl-2 to investigate the interplay between the nervous system and white adipose tissue (WAT).

**Methodology:**

Changes in the architecture and innervation of WAT were compared between wildtype, Nscl2−/−, ob/ob and Nscl2−/−//ob/ob mice using morphological methods, immunohistochemistry and flow cytometry. Metabolic alterations in mutant mice and in isolated cells were investigated under basal and stimulated conditions.

**Principal Findings:**

We found that Nscl-2 mutant mice show a massive reduction of innervation of white epididymal and paired subcutaneous inguinal fat tissue including sensory and autonomic nerves as demonstrated by peripherin and neurofilament staining. Reduction of innervation went along with defects in the formation of the microvasculature, accumulation of cells of the macrophage/preadipocyte lineage, a bimodal distribution of the size of fat cells, and metabolic defects of isolated adipocytes. Despite a relative insulin resistance of white adipose tissue and isolated Nscl-2 mutant adipocytes the serum level of insulin in Nscl-2 mutant mice was only slightly increased.

**Conclusions:**

We conclude that the reduction of the innervation and vascularization of WAT in Nscl-2 mutant mice leads to the increase of preadipocyte/macrophage-like cells, a bimodal distribution of the size of adipocytes in WAT and an altered metabolic activity of adipocytes.

## Introduction

The regulation of energy balance is controlled by a complex system that allows the brain to sense and integrate various signals in order to elicit suitable changes in food intake and energy expenditure. A failure of the CNS control of food intake will result in an increase of the mass of WAT of an organism by a combination of increasing adipocyte cell size and number. The increase in WAT mass is not only caused by expansion of adipocytes but also by differentiation of fibroblast-like preadipocytes that are already determined for an adipocyte fate. Preadipose cell lines and primary cultures of preadipocytes are committed solely to the adipocyte lineage and differentiate either spontaneously or under the influence of adipogenic hormones such as IGF-I and glucocorticoids into mature adipocytes [1]–[4]. Adipose tissue is not only the main energy reservoir of the body but a complex organ in which adipocytes, connective tissue matrix, nerve tissue, stromal vascular cells and immune cells function as an integrated unit. Molecules secreted by adipocytes include adipocytokines, e.g. leptin, adiponectin, resistin, interleukin 6 (IL-6) but also IGF-1, adenosine, plasminogen activator inhibitor, and TNF-α. These factors are essential components of the peripheral signals that control regulatory processes in the hypothalamus affecting energy homeostasis and reproduction [5]–[7].

The majority of pathways that control feeding behavior converge on the hypothalamus [8] although several “peripheral” anabolic and catabolic hormones act at various sites within the central nervous system. The integration and interpretation of incoming signals involves several hypothalamic transcription factors including the basic helix-loop-helix protein Nscl-2 (Neurological stem cell leukemia). Nscl-2 is also known as Nhlh2 (nescient helix-loop-helix 2) according to the HUGO and NCBI nomenclature. Nscl-2 and the closely related NSCL-1 are expressed in large areas of the developing central and peripheral nervous system [9], [10] with Nscl-2 being expressed within the paraventricular nucleus (PVN), arcuate nucleus (ARC), and in neurons of lateral, ventromedial and dorsal medial hypothalamus of adult mice [11]. Nscl-2 mutant mice show an adult onset of obesity [12] and infertility, which is at least in part caused by disrupted migration of developing GnRH-1 neurons [13]. Adolescent Nscl-2 mutant animals show reduced physical activity while adult obese Nscl-2 mutant mice are characterized by both increased food intake and reduced voluntary physical activity [14]. Neither NSCL-1 nor Nscl-2 are expressed outside the nervous system as demonstrated by numerous expression studies and by the use of LacZ-knock-in reporter mice [10].

So far, the knowledge about a direct control of the metabolic activity of adipocytes or their differentiation by CNS-derived signaling pathways is limited [3]. Similarly, no clear correlation between peripheral innervation and adipocyte differentiation or metabolic activity has been established and genetic systems to study such effects were missing. On the other hand, it has been postulated that sympathetic nerves are involved in the control of lipolysis [15], [16] and it has been shown that sympathetic denervation of WAT triggers an increase in fat cell number although it is unknown whether this is due to preadipocyte proliferation or maturation of existing preadipocytes [17]. The nervous system might also affect the white adipose tissue indirectly by targeting blood vessels that influence adipocytes [18]. However, a general link between peripheral innervation and the degree of microvascularization of white adipose tissue has not been disclosed yet.

In the current study we have employed Nscl-2 knockout mice to investigate the interplay between the nervous system and WAT. We found that Nscl-2 knockout mice show a reduced peripheral innervation and microvascularization, which correlated with an accumulation of preadipocyte/macrophage-like cells and heterogeneity of adipocyte sizes.

## Results

### Nscl-2 mutant mice suffer from reduced innervation and vascularization of WAT

**Figure 1:**
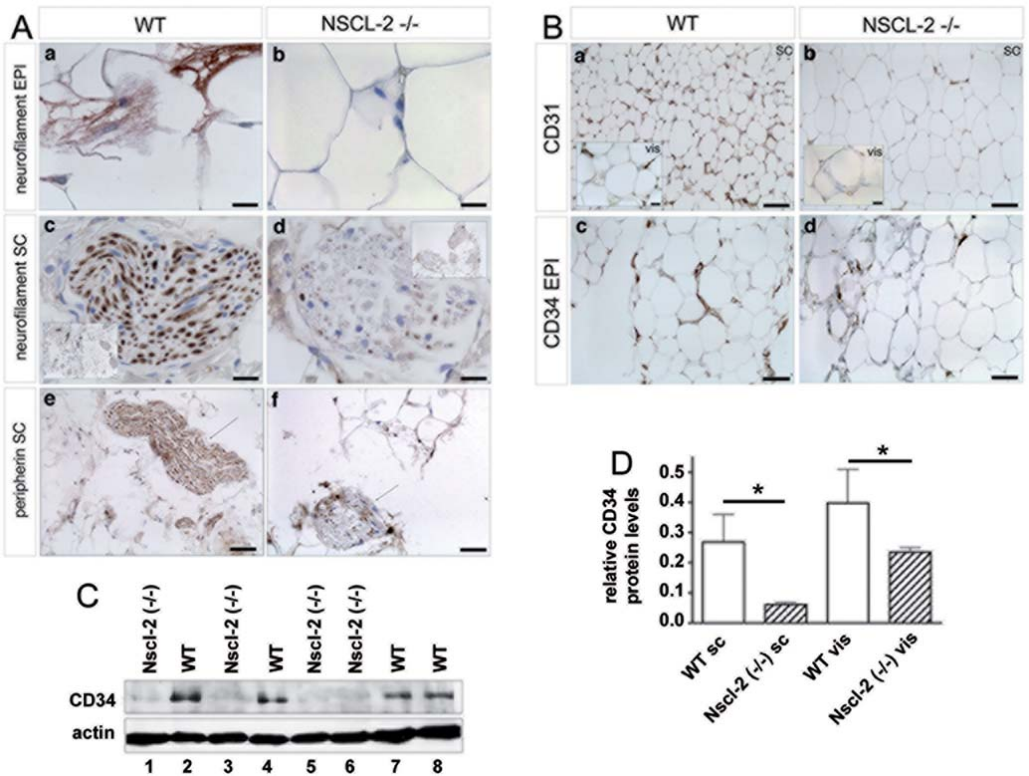
Reduced innervation and vascularization of WAT in Nscl-2 deficient mice. (A) Paraffin sections of WAT from wild-type (WT) and Nscl-2 −/− mice stained with an antibody against neurofilament (a–d) and peripherin (e, f). (a, b) sections through epididymal fat; (c–f) sections through nerve fascicles within subcutaneous fat tissue. Scale bars in (a–d) 15 µm, in the small pictures in (c, d) 100 µm and in (e, f) 50 µm. (B) Reduced vascularization of subcutaneous (SC) and epididymal (vis) fat tissue of Nscl-2 mutants. Immunostainings for endothelial cell markers CD31 (a, b) and CD34 (c, d). Scale bars: 50 µm and in small pictures in (a, b) 15 µm. (C) Western blot analysis of CD34 protein expression in epididymal and paired subcutaneous inguinal adipose tissue of wild-type and Nscl-2 knockout mice. Actin was used as a loading control. (D) Quantification of Western blot analysis of CD34 protein expression in epididymal and paired subcutaneous inguinal adipose tissue of wild-type and Nscl-2 knockout mice. Actin was used as a loading control.

**Figure 2:**
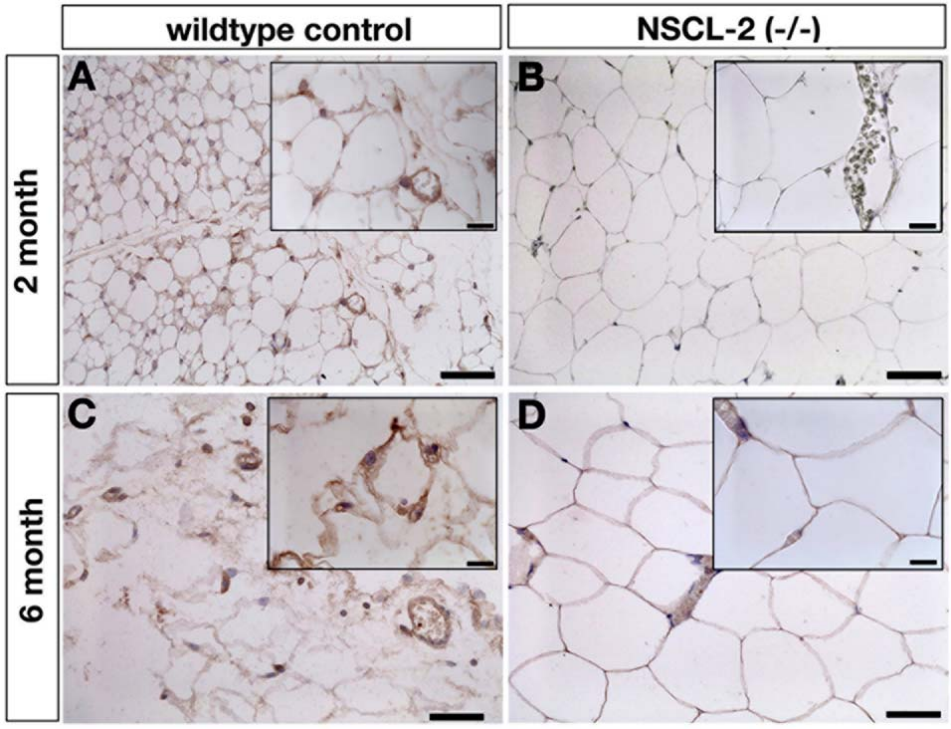
Reduced innervation of WAT in Nscl-2 deficient mice at different postnatal stages. Paraffin sections of WAT from wild-type (A, C) and Nscl-2 −/− mice (B, D) at 2 (A, B) and 6 month (C, D) of age stained with an antibody against peripherin. Sections were taken from epididymal fat tissues. Note the reduction of peripherin positive nerves in Nscl-2 mutants. Scale bars 100 µm; in small pictures 15 µm.

Deletion of Nscl-2 leads to a complex neurological phenotype that comprises infertility and an adult-onset of obesity. These defects are most likely the result of malfunctions of the regulatory network within the CNS, which control genes important to energy expenditure, and more specifically voluntary physical activity of animals [14]. We wondered whether disruption of the Nscl-2 gene did also affect development of the PNS since Nscl-2 is strongly expressed in the dorsal root ganglia and the autonomic nervous system [10], [19]. For our analysis we concentrated on WAT since morphological analysis of different tissues revealed pathological changes in WAT, which were apparent ***before*** the animals became obese (see below). We detected a strong reduction of the expression of neurofilament, a marker for sensory neurons [20], in epididymal and subcutaneous adipose tissue of Nscl-2 mutants (Fig. 1A). Small nerve fibers that are normally easily detected within the adipose tissue were absent together with a strong reduction of neurofilament staining within funiculi and larger nerve fascicles. In the subcutaneous adipose tissue the funiculi of Nscl-2 (−/−) mice were almost devoid of neurofilament (Fig. 1Aa–d). We also investigated the presence of unmyelinated fibers in adipose tissues, which include postganglionic fibers of the autonomic nervous system (sympathetic and parasympathetic). Unmyelinated fibers are marked by expression of peripherin while neurofilament is specifically expressed in myelinated fibers [20]. We found a massive decrease of the number of peripherin-positive fibers in epididymal and subcutaneous adipose tissue of Nscl-2 mutants at 8, 16 and 24 weeks after birth (Fig. 1Ae, f, Fig. 2). Normally, neurofilament and peripherin positive axons are embedded into streets of connective tissue along the capillaries and recent evidence suggests an intensive crosstalk between the vascular and the nervous system [21]. Hence, we also wanted to know whether the reduction of nerve fibers might affect formation of the microvasculature in adipose tissue of Nscl-2 mutant mice. Immunohistochemical staining with antibodies against the vascular markers CD31 and CD34 revealed a strong decrease of microvasculature formation in Nscl-2 mutant mice (Fig. 1B) both in subcutaneous and epididymal fat tissue. Quantitative assessment of the expression of CD34 by Western blot analysis disclosed a decrease of CD34 expression of 77±1% in subcutaneous and of 41±2% in epididymal fat tissue (Fig. 1C, D), which corresponded to the reduced presence of microvasculature observed after immunohistochemical staining.

### Increased expression of leptin precedes obesity in Nscl-2 mutant mice

**Figure 3:**
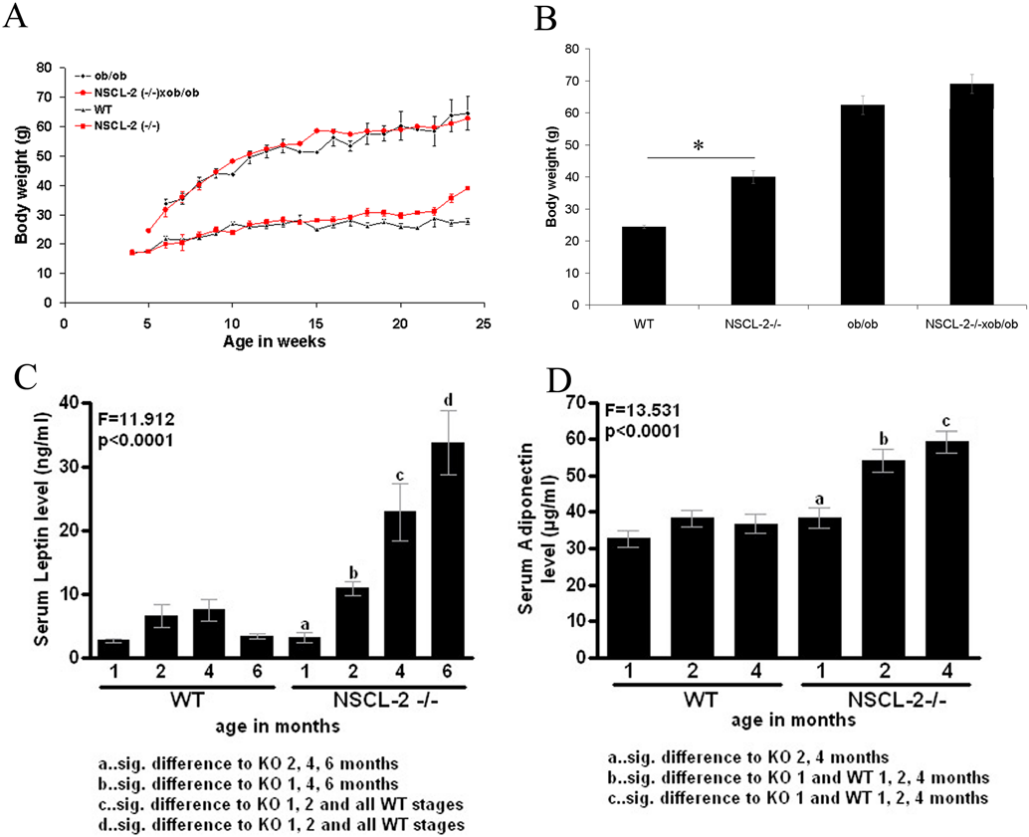
Increased expression of leptin precedes abnormal gain of weight in Nscl-2 mutant mice. (A) Significant differences in the body weight of WT and Nscl-2 (−/−) mice occur only post-puberty after 12–15 weeks of age (lower two graphs). Nscl-2 (−/−)×ob/ob and ob/ob mutant mice show a similar increase of body weight during postnatal growth (upper two graphs). (B) Body weight of 6–8 month old Nscl-2 (−/−), Nscl-2 (−/−)×ob/ob, ob/ob, and wild type male mice (F = 53.33; dF = 3; p<10^−4^). (C) Serum leptin levels are massively elevated in Nscl-2 (−/−) mice at 2, 4 and 6 month (F = 11.91; dF = 7; p<0.001). Note that the increase of serum levels of leptin in Nscl-2 mutant mice precedes the abnormal gain of weight. (D) Serum adiponectin levels are elevated in Nscl-2 (−/−) mice at 2, and 4 month compared to wildtype control animals (F = 13.53; dF = 7; p<0.0001).

**Figure 4:**
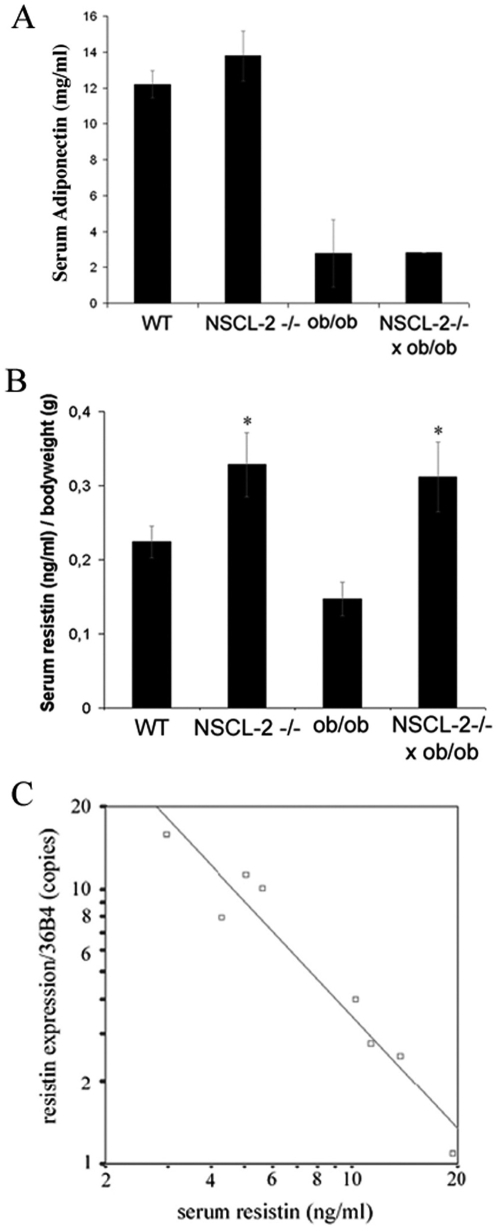
Nscl-2 mutant mice show increased serum adiponectin and resistin levels. (A) High serum adiponectin levels in adipose Nscl-2 mutant mice. Adiponectin levels in 6–8 months old WT control (n = 4), Nscl-2 (−/−) (n = 9), ob/ob (n = 3), and Nscl-2 (−/−)×ob/ob (n = 1). (B) Increased serum resistin levels in Nscl-2 and Nscl-2/leptin compound mutant mice. Serum resistin level were divided by body weights of WT (n = 23), Nscl-2 (−/−) (n = 15), ob/ob (n = 14) and Nscl-2 (−/−)×ob/ob mice (n = 8). Asterisks indicate significant higher weight matched serum resistin level in Nscl-2 (−/−) (F = 6.3; dF = 3; p<10^−4^). (C) Serum resistin levels are inversely proportional to mRNA expression of resistin in WAT (R^2^ = 0.89; p = 0.004). WT (n = 2), Nscl-2 (−/−) (n = 2), ob/ob (n = 2) and Nscl-2 (−/−)×ob/ob (n = 2).

Since our morphological analysis revealed a reduction of innervation and vascularization in Nscl-2 well before the mutant mice became obese we wondered whether Nscl-2 mutant WAT might also show an aberrant release of adipokines prior to the abnormal gain of weight. To address this question we first monitored the increase of body weight in our strain of Nscl-2 mutant mice. We found that Nscl-2 mutant mice show a normal birth weight and did not gain excessive weight until after puberty. After puberty, which has been reported for C57 mice to occur between 4 weeks (vaginal opening) and 8.5 weeks (cyclicity) [22], body fat increased noticeably (Fig. 3A) eventually resulting in a body weight of Nscl-2 (−/−) mice that was 34% higher compared to WT mice (32.64±0.63 g versus 24.42 g±0.53 g) at the age of 25 weeks (Fig. 3B). This observation corroborated previous reports [14], [23], which were generated using a different strain of Nscl-2 mutant mice. Interestingly, serum concentrations of leptin and adiponectin in Nscl-2 mutant mice were already significantly elevated at 8 weeks, at a time when the body weight was not significantly different between mutant and wildtype animals (Fig 3C, D). After puberty the leptin level increased further and reached a peak at 24 weeks (Fig 3C). We also detected a marked elevation in the serum concentrations of adiponectin and resistin in 6–8 month old NSLC-2 (−/−) mice (Fig. 4). The high levels of leptin in Nscl-2 knockout mice suggested that these mice are resistant to leptin signaling. Indeed, peripherally administration of leptin with osmotic mini pumps over a time period of 14 days did not result in a significant reduction of the body weight of Nscl-2 mutants compared to C57Bl/6 controls (data not shown). We concluded that the increase of serum leptin and adiponectin levels was caused by changes in the WAT such as decreased innervation and vascularization that occurred before onset of obesity.

### Nscl-2 knockout mice show increased numbers of preadipocyte/macrophage-like cells and a bimodal distribution of the size of adipocytes in WAT

**Figure 5:**
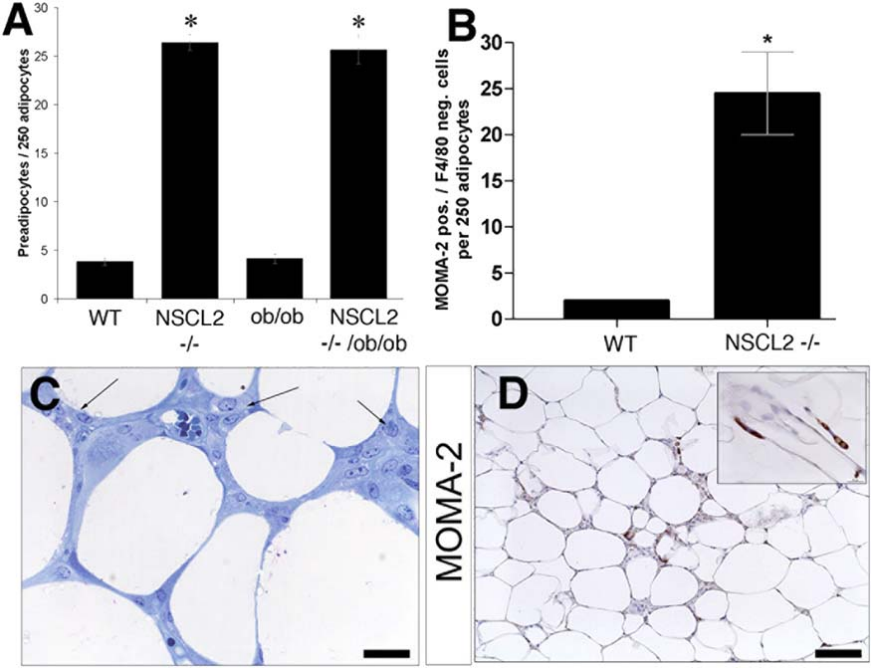
High number of preadipocyte/macrophage-like cells in the adipose tissue of Nscl-2 (−/−) and Nscl-2 (−/−)×ob/ob compound mutant mice. (A) Number of preadipocyte/macrophage-like cells per 250 adipocytes (F = 250.71; dF = 3; p<10^−4^; 4 mice per group). (B) Number of MOMA-2 positive but F4/80 negative cells per 250 adipocytes. The difference between MOMA-2 and F4/80 positive cells corresponds to adipocyte precursors (p = 0.038). (C) Richardson-stain of a section of epididymal adipose tissue from 6 months old male double mutant mouse. Arrow indicates groups of preadipocyte/macrophage-like cells between fat cells (scale bar: 25 µm). (D) MOMA-2 staining of paraffin embedded epididymal adipose tissue of a Nscl-2 (−/−) mouse (scale bar: 100 µm). The inlet shows a typical staining of MOMA-2 (scale bar: 15 µm).

**Figure 6:**
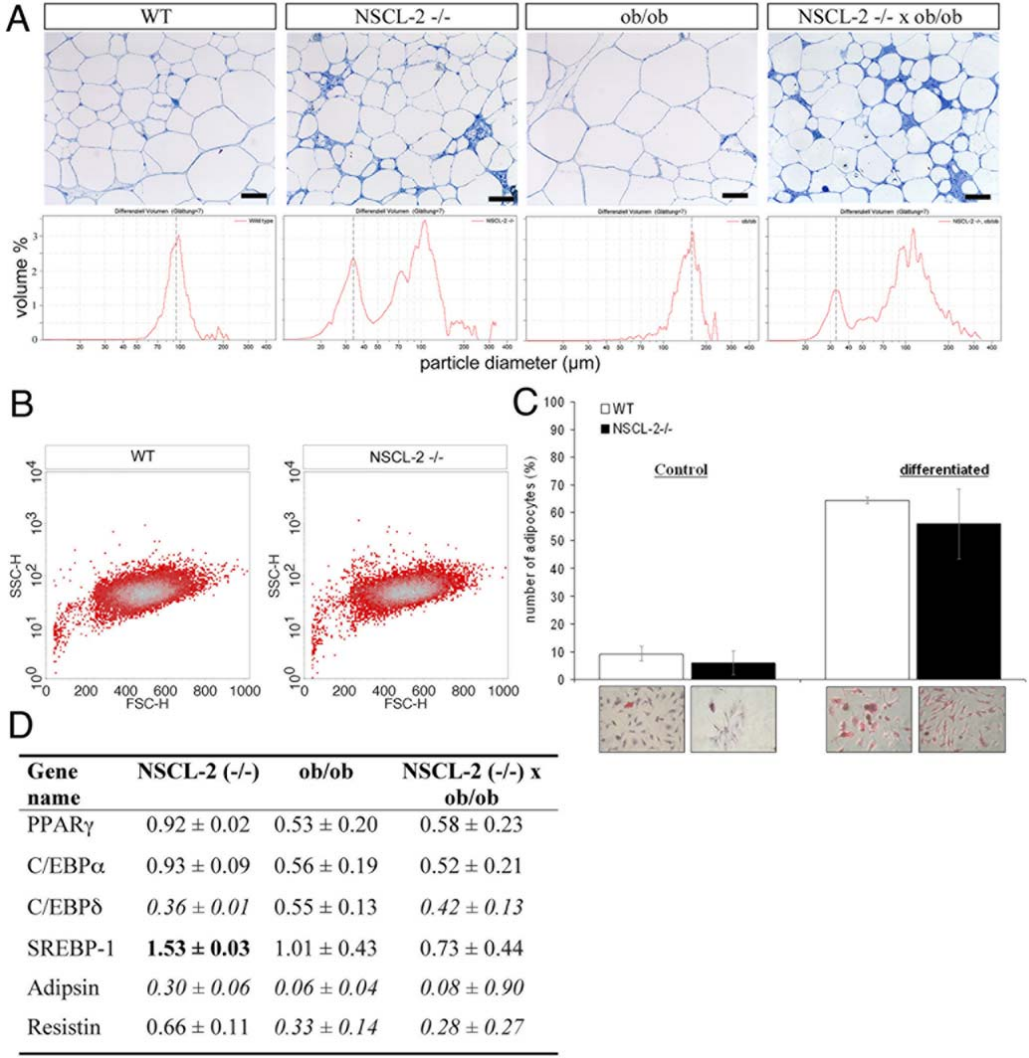
Bimodal distribution of adipocytes in Nscl-2 (−/−) mice and normal *in vitro* differentiation of isolated Nscl-2 mutant preadipocytes. (A) Semithin sections (Richardson stain) of epididymal adipose tissue from 6 month old male animals. Histograms indicate the size distribution of isolated adipocytes from different strains. Scale bars: 100 µm. (B) FACS analysis (sideward versus forward scatter) of preadipocytes after 1 day in culture reveals a general uniformity in cell size and granularity. (C) Normal *in vitro* differentiation of isolated Nscl-2 mutant preadipocytes. Differentiated, lipid drop containing cells were identified by oil red staining. (D) Relative mRNA expression level of adipocyte differentiation related genes and adipocytokines expressed in epididymal WAT determined by quantitative Real-Time PCR (mean values, n≥3). Values in bold indicate changes >150% compared to controls, values in italic indicate changes <50% of compared to controls. Relative expression level = (GeneX^mutant^/36B4^mutant^)/ (GeneX^WT^/36B4^WT^).

Previously, it had been demonstrated that sympathetic denervation of WAT triggers an increase in fat cell number, which might be due to preadipocyte proliferation or maturation of existing preadipocytes [17]. We therefore studied more closely the architecture of white adipose tissue of wildtype, Nscl-2 (−/−) mice using semithin sections. Additionally we also characterized ob/ob, and Nscl-2 (−/−)×ob/ob compound mutant mice to study whether Nscl-2 deficiency changed the fat cell phenotype of the leptin deficient ob/ob strain. Adipose tissue of wild type and ob/ob mutant mice contained less than 5 preadipocyte/macrophage-like cells per 250 adipocytes while Nscl-2 (−/−) mice showed a 7-fold increase of cells of the preadipocyte/macrophage-like lineage in WAT at 6 months of age (F = 250.71; dF = 3; p<10^−4^; Fig. 5A). The arrows in Figure 5C indicate groups of small preadipocyte/macrophage-like cells between mature adipocytes. To further characterize this group of small cells with the focus on identification of preadipocytes we used a combination of MOMA-2 and F4/80 antibodies. MOMA-2 (macrophage and monocyte antibody-2) is expressed by macrophages and preadipocytes including the 3T3-L1 preadipocyte cell line while the F4/80 antibody specifically detects macrophages but not preadipocytes such as 3T3-L1 cells. Hence, the difference between MOMA-2 and F4/80 positive cells corresponds to adipocyte precursors [24]. We counted 2 MOMA-2 pos. / F4/80 neg. cells per 250 adipocytes in WT but 24±4 per 250 adipocytes in Nscl-2 (−/−) mice, which exactly corresponded to the number of preadipocyte/macrophage-like cells found in semithin sections of Nscl-2 mutant epididymale tissue (Fig. 5A, B). We also accessed the size of adipocytes in wild type, Nscl-2 (−/−), ob/ob, and Nscl-2 (−/−)×ob/ob compound mutant mice using flow cytometry. We detected two groups of adipocytes: one small group with adipocytes about 35 µm in diameter and a second group with adipocytes larger than 100 µm. While virtually no small adipocytes were found in the adipose tissue of wild type and ob/ob mutant mice huge numbers of small cells were detected in Nscl-2 (−/−) and Nscl-2 (−/−)×ob/ob compound mutant mice resulting in a bimodal distribution of the size of adipocytes (Fig. 6A).

To determine whether the high number of preadipocyte/macrophage-like cells in Nscl-2 deficient mice resulted from cell autonomous differentiation defects we isolated the stromal vascular fraction (SVF) containing preadipocyte/macrophage-like cells from WT (n = 4) and Nscl-2 −/− animals (n = 3) and subjected them to differentiation *in vitro*. Flow cytometry based characterization of these cells revealed a general uniformity in cell size and granularity after 1 day in culture (Fig. 6B). Two days later confluent, cultured adipocytes were induced to differentiate by addition of dexamethasone, insulin and IBMX. 9 days after induction we counted the formation of mature adipocytes as identified by oil red staining (Fig. 6C). Nscl-2 (−/−) adipocytes differentiated readily giving rise to 56%±12.5% oil red stained cells as compared to 64%±1.2% oil red stained WT cells indicating a normal differential potential of Nscl-2 mutant preadipocytes. No significant reduction of the expression of PPARγ, C/EBPα, C/EBPδ and SREBP-1, which play decisive roles in the differentiation of adipocytes [3], were detected in Nscl-2 mutant epididymal adipose tissue although a slight reduction of PPARγ, C/EBPα in ob/ob and in Nscl-2 (−/−)×ob/ob compound mutant mice was noted (Fig. 6D). Taken together our results suggest that the cell differentiation potential of Nscl-2 mutant preadipocytes is not impaired.

### Adipocytes from Nscl-2 mutant mice show increased basal glucose uptake and lipolysis but decreased insulin sensitivity

**Figure 7:**
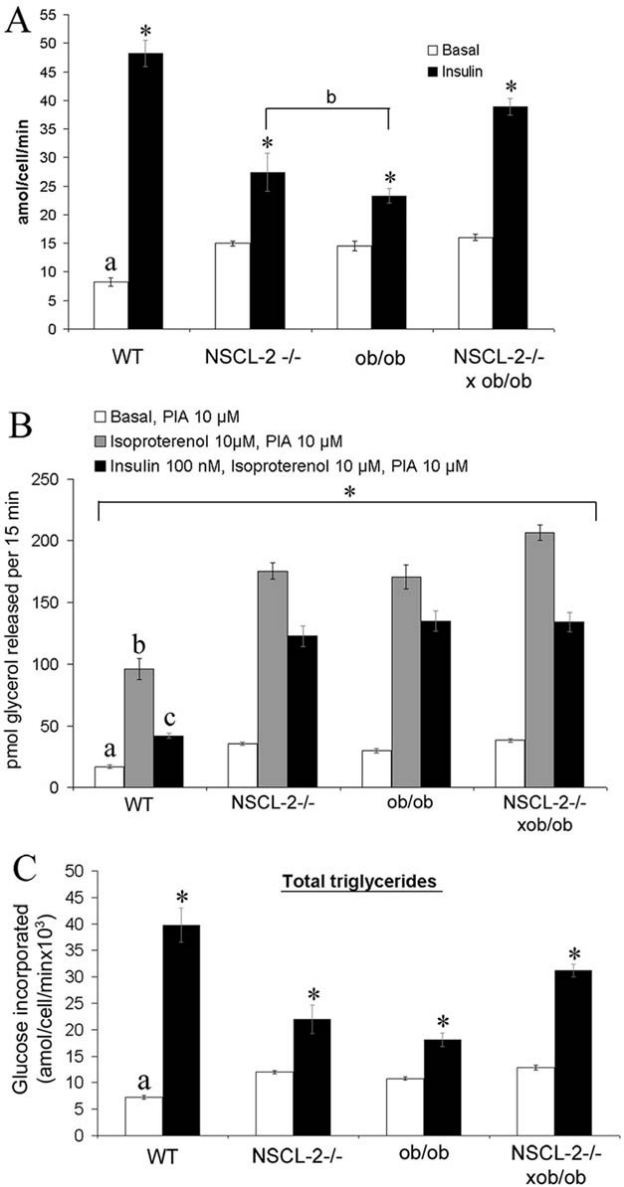
Impaired glucose metabolism and decreased insulin sensitivity in isolated adipocytes of Nscl-2−/−, Nscl-2−/−×ob/ob mice and controls. (A) Basal and insulin stimulated U-[^14^C]glucose uptake in isolated adipocytes from 6 moth old male WT, ob/ob, Nscl-2 (−/−) mice (n = 4) and double mutant mice (n = 3). Insulin stimulated glucose uptake was significant higher in all groups compared to basal levels (p<0.005). a: significant difference between WT basal and basal of all others (F = 28.17; dF = 3; p<10^−4^), b: significant difference between insulin stimulated glucose uptake by Nscl-2 −/− and ob/ob versus WT and double mutant adipocytes (F = 24.22; dF = 3; p<10^−4^). (B) Lipolysis in isolated adipocytes. Basal rates of lipolysis in the presence of 10 µM PIA (N6-[R-(-)-1-methyl-2-phenyl]adenosine) were compared to stimulated lipolysis in the presence of 100 µM isoproterenol and to a treatment with 100 nM insulin, 15 min prior to the addition of 100 µM isoproterenol (WT: F = 60.8; dF = 2; p<10^−4^. Nscl-2 (−/−): F = 131.8; dF = 2; p<10^−4^. ob/ob: F = 97.1; dF = 2; p<10^−4^. Nscl-2 (−/−)×ob/ob: F = 212.1; dF = 2; p<10^−4^). a: significant difference of WT basal glycerol release versus basal level of all others (F = 44; dF = 3; p<10^−4^); b: significant difference of WT maximum glycerol release versus maximum release level of all others (F = 32; dF = 3; p<10^−4^); c: significant difference of WT 100 nM insulin+100 µM isoproterenol stimulated glycerol release versus levels of all others (F = 42; dF = 3; p<10^−4^). (C) Glucose metabolism into triglycerides (TG) in isolated adipocytes at 5 mM glucose in the absence (basal) or presence of 80 nM insulin (asterisks indicate significant differences). a: significant difference between WT basal and basal of all others (F = 46.4; dF = 3; p<10^−4^).

To study whether aberrant innervation and vascularization caused by the lack of Nscl-2 had an impact on the metabolic activity of WAT we investigated the glucose and/or lipid metabolism of isolated adipocytes derived from Nscl-2 (−/−) and Nscl-2 (−/−)×ob/ob compound mutant mice. Basal glucose uptake in adipocytes from Nscl-2 (−/−), ob/ob, and double mutant mice was significantly higher compared to wildtype adipocytes (Fig 7A). However, stimulated glucose uptake (after treatment with 80 nM insulin) was strongly mitigated in Nscl-2 (−/−) and ob/ob adipocytes compared to wildtype controls indicating reduced insulin sensitivity (Fig. 7A). Analysis of glycerol release revealed an increased basal level of lipolysis in Nscl-2 (−/−), ob/ob, and double mutant adipocytes compared to wildtype controls (Fig. 7B). Moreover, ß-adrenergic stimulation led to a strong stimulation of lipolysis in Nscl-2 (−/−)×ob/ob compound mutants, which was nearly twice as high as in control cells (Fig. 7B). Inhibition of isoproterenol-stimulated glycerol release by insulin resulted in a decrease of glycerol release of 56.4%±13.9% in wildtype, 29.9%±10.5% in Nscl-2 deficient, 20.7%±11.8% in ob/ob adipocytes, and 35%±8.8% in compound mutant adipocytes again indicating an alleviation of the insulin resistance in double mutant adipocytes. We also measured the metabolic contribution of radioactively labeled glucose to lipogenesis (incorporation into total triglycerides) under basal and insulin stimulated conditions. Insulin treatment led to a better utilization of glucose in all groups (Fig. 7C).

### Impaired insulin sensitivity but normal glucose tolerance in Nscl-2 (−/−) mice

**Figure 8:**
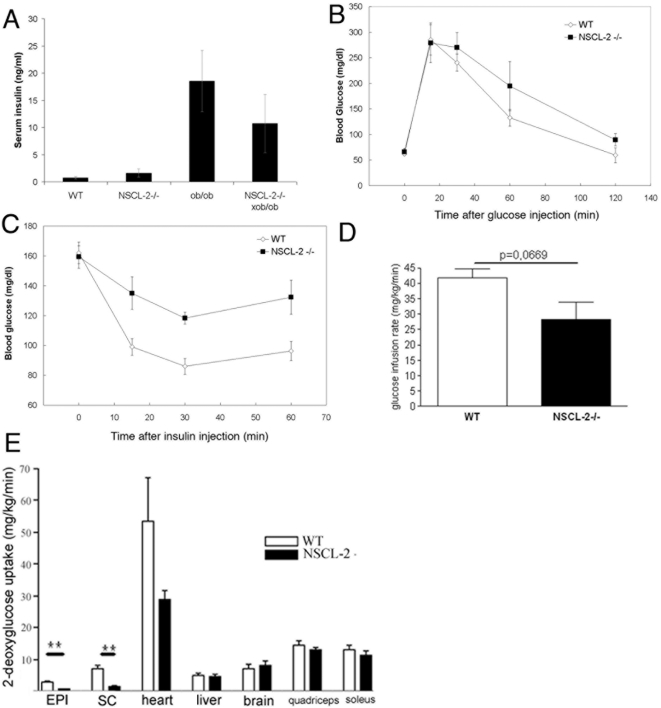
Metabolic parameters and insulin sensitivity in Nscl-2 −/− and WT mice during hyperinsulinemic euglycemic clamp. (A) *Ad libitum* serum insulin level measured at 9 am in WT controls (n = 8), ob/ob mice (n = 12), Nscl-2 mutants (n = 3) and double mutants (n = 3). (B) Glucose tolerance test of 6 months old female WT and Nscl-2 (−/−) mice (n = 4). (C) Insulin tolerance test. Means at 15, 30 and 60 minutes are significant higher in Nscl-2 mutants compared to WT mice, (at 15 min: p = 0.013; at 30 min: p = 0.0014; at 60 min: p = 0.017). (D) The glucose infusion rate during a hyperinsulinemic-euglycemic clamp was decreased in Nscl-2 knockout mice compared to C57/Bl6 wild-type mice (n = 6). (E) Insulin stimulated 2-deoxyglucose uptake in vivo in different tissues during the clamp. Nscl-2 −/− mice show specific insulin resistance in WAT (EPI: 70% reduction (p = 0.003); SC: 79% reduction (p = 0.010).

The massive obesity of Nscl-2 mutant mice and the insulin resistance of Nscl-2 deficient adipocytes suggested that Nscl-2 mutants might suffer from type II diabetes. Interestingly however, the serum insulin levels of Nscl-2 mutant mice were virtually normal while ob/ob mice, which are a model of Type II diabetes [25], showed a massive increase in serum insulin concentrations (Fig. 8A). Moreover, Nscl-2 (−/−)×ob/ob compound mutants displayed a reduction of serum insulin concentration when compared to ob/ob mice of the same age, sex and body weight (Fig. 8A). While glucose tolerance tests did not show significant differences between WT and Nscl-2 (−/−) mice (Fig. 8B) we observed a reduced insulin sensitivity of Nscl-2 (−/−) mutant mice in insulin tolerance tests. Injections of 1 U insulin/kg body weight in wild type and Nscl-2 (−/−) animals caused a more pronounced drop of glucose levels in wild type compared to mutant animals 15, 30 and also 60 min after insulin treatment (p<0.05, Fig. 8C).

The effect of the deletion of Nscl-2 on insulin action was further characterized in a 2 h hyperinsulinemic-euglycemic clamp. We detected a reduction of the steady state glucose infusion rate required for maintenance of euglycemia in Nscl-2 (−/−) mice by 32% (Fig. 8D) confirming the insulin resistance of Nscl-2 deficient mice. To measure time-dependent insulin-stimulated glucose uptake into different tissues we injected a bolus of 100 µCi 2-[1-^14^(C)]-Deoxy-D-glucose at the end of the hyperinsulinemic-euglycemic clamp. Glucose uptake of Nscl-2 mutant animals was only reduced in adipose tissue but not in striated muscles. A certain reduction was also noted in the heart although this result was statistically not significant due to variations in glucose uptake of WT mice (subcutaneous fat: 78%, epididymal fat: 70%; p<0.01; Fig. 8E) [26], [27].

## Discussion

So far no clear link between peripheral nerve activity and architecture and/or metabolic activity of adipose tissue had been established although accumulating evidence suggested that disturbed local interactions of peripheral nerves and adipose tissue might affect the activity of adipocytes and the control of preadipocyte differentiation [28]. Nscl-2 mutant mice show a striking reduction of the innervation and vascularization of WAT, which might lead to the increase of preadipocyte/macrophage-like cells, the bimodal distribution of the size of adipocytes in WAT and to the altered metabolic activity of adipocytes. We do not believe that the elevated food intake and reduced physical activity of Nscl-2 mutant mice is responsible for the WAT phenotype since comparable changes have not been found in other models of obesity, excessive food intake, and physical inactivity [29]. In addition the changes in the architecture of WAT in Nscl-2 developed before the mutant became obese. Similarly, we observed an increase of the serum concentrations of leptin and adiponectin before Nscl-2 mutant mice gained excessive weight clearly indicating that the changes in the architecture and/or metabolic activity of adipose tissue were not directly caused by the obesity, which develop later during life in Nscl-2 mutants.

Interestingly, the accumulation of preadipocytes is a rather dominant phenotype, which prevailed even in Nscl-2/leptin compound mutant mice indicating that leptin most probably is not involved in this process. Currently, we favor the hypothesis that the massive reduction of peripheral innervation and vascularization in Nscl-2 mutant WAT is the primary cause for metabolic changes in adipocytes and the morphological alterations of the WAT. This statement is primarily supported by the exclusive expression of Nscl-2 in the nervous system as well as by the reduced innervation of WAT and increased release of adipokines before puberty, preceding the onset of obesity. However, we cannot completely exclude the possibility that other host-derived signals contribute to the WAT phenotype. In fact, we detected a marked elevation in the serum concentrations of numerous adipokines such as leptin as well as resistin and adiponectin, which might have an effect on the differentiation of preadipocytes. The high leptin serum level result in an early leptin resistance of Nscl-2 knockout mice. In particular resistin has been described to inhibit preadipocyte differentiation in vitro [30] while adiponectin promotes cell proliferation and differentiation from preadipocytes into adipocytes [31]. The massive increase of the concentration of various adipokines might enhance the heterogeneity of adipocytes and contribute to the accumulation of high numbers of preadipocytes.

Currently, we do not know whether the massive reduction of microvasculature in the WAT of Nscl-2 mutants contributes to the phenotype. Although it is well known that the neuronal and the vascular system share similar signaling cues, the concept of a neurovascular link that recognizes the crosstalk between the vascular and the nervous system is relatively new [21]. Nscl-2 mutant mice represent the first model, which displays a clear correlation between reduced innervation and reduction of vascularization in the WAT.

The primary defect in Nscl-2 mutant mice lays in the nervous system but not in adipose tissue. Yet, adipocytes isolated from Nscl-2 mutant mice are insulin-resistant indicating that the metabolic changes that develop in mutant mice led to a relatively stable phenotype of adipocytes, which maintain these features in vitro for some time. Although the serum concentration of insulin was relatively low in Nscl-2 mutant mice, which argues against a massive insulin resistance of insulin responsive cells, it is evident that Nscl-2 mutant adipocytes have acquired properties in vivo that led to an impaired glucose transport in vitro. The deficiency of adipocytes was restricted to insulin stimulated glucose uptake but did not affect the basal glucose uptake rate demonstrating that the basic glucose uptake machinery was intact. It was surprising to learn that Nscl-2 mutant mice show a normal glucose tolerance and only a moderate decrease of insulin sensitivity despite the insulin resistance of isolated adipocytes. This apparent paradox questions the role of white adipose tissue in overall glucose homeostasis [32]. Interestingly, mice with an adipose tissue specific insulin receptor knockout (FIRKO mice) show a normal glucose tolerance and insulin sensitivity and are protected against obesity-related glucose intolerance [33], which resemble the Nscl-2 phenotype to certain degree. On the other hand, various syndromes of lipodystrophy, insulin resistance associated with obesity [34], and insulin resistance of mice with a fat-specific knockout of GLUT4 [35] demonstrate an important role of fat tissue in overall glucose homeostasis. Despite the important role of adipocytes and WAT, the capacity of other tissues such as the skeletal muscle to clear glucose seemed to compensate for the impaired insulin response of adipocytes in Nscl-2 mutant mice. The relative increase of adiponectin that we detected in Nscl-2 (−/−) mice might also contribute to improved insulin signalling in non-adipose tissue and to normal serum glucose levels in vivo. Such effects would be masked in tissue culture experiments where isolated adiopocytes are analyzed. The increase in adiponectin levels in Nscl-2 (−/−) mice was rather unexpected since adiponectin levels are usually inversely correlated to percent body fat resulting in the reduction of adiponectin concentrations in obese animals (see [36] for a review). It is tempting to speculate that the unusual morphology of WAT in Nscl-2 mutant mice, which resulted from the accumulation of preadipocyte/macrophage-like cells and the bimodal distribution of the size of adipocytes in WAT, was responsible for this surprising finding.

**Figure 9:**
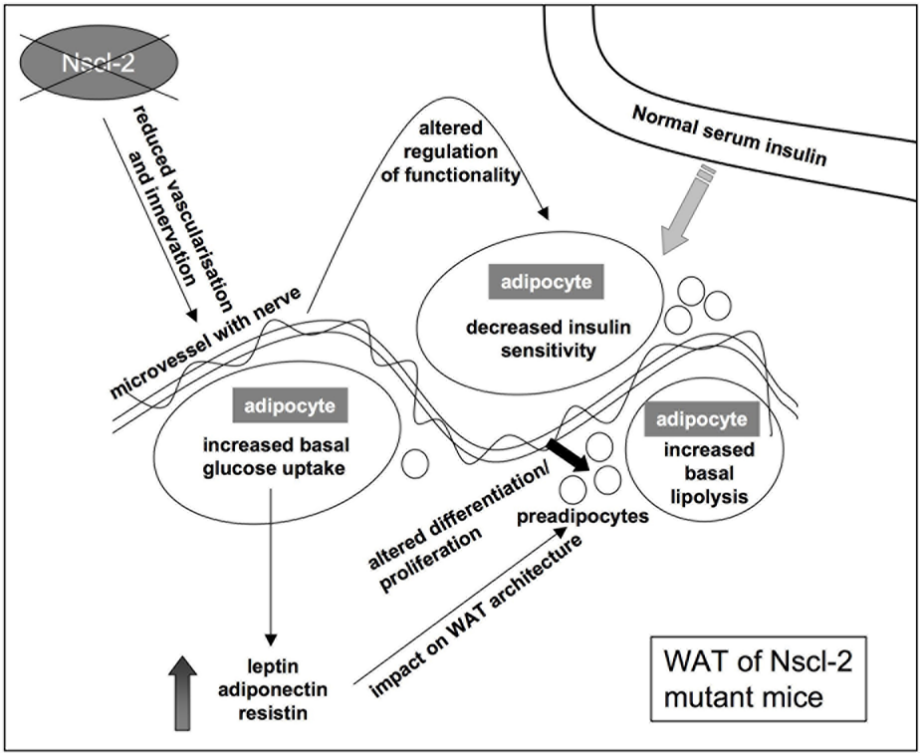
Putative model of the impact of Nscl-2 controlled mechanisms on WAT. The lack of Nscl-2 results in reduced innervation and vascularization of WAT, which most likely leads to accumulation of preadipocyte/macrophage-like cells and the bimodal distribution of the size of adipocytes in WAT. The altered functionality of adipocytes is reflected by decreased insulin sensitivity, increased basal glucose uptake, and increased basal lipolysis. The decreased insulin sensitivity of isolated adipocytes seems to be compensated by other tissues, which might explain the relatively normal serum insulin concentrations.

Taken together, we have uncovered a hitherto unknown regulatory circuit that originates from the deficiency of the neuron specific transcription factor Nscl-2 with consequences for innervation of adipose tissue, which leads to accumulation of preadipocytes in vivo. So far the effects of Nscl-2 on fat cell metabolism had been solely explained by alterations in the CNS. Although it is undisputable that the increased food intake and reduced physical activity of Nscl-2 mutant mice are the primary causes of adiposity in Nscl-2 mutant mice it now becomes clear that Nscl-2 governs additional pathways that affect the WAT. Fig. 9 summarizes our findings and presents a putative model for the impact of Nscl-2 controlled mechanisms on WAT.

## Materials and Methods

### Ethics Statement

The investigation conforms to the *Guide for the Care and Use of Laboratory Animals* published by the US National Institutes of Health (NIH Publication No. 85-23, revised 1996) and was approved by the local authorities of the state of Sachsen-Anhalt, Germany as recommended by the responsible local animal ethics review board.

### Mice and metabolic studies

The generation of Nscl-2 knockout mice has been described previously [13]. Nscl-2 knockout mice were backcrossed for >10 generation to C57BL/6J mice and maintained on the same background. Six to eight weeks old heterozygous Nscl-2 (+/−) mice bred to heterozygous C57BL/6J Lep^ob^ mice of purchased from the Jackson Laboratory to generate double heterozygous Nscl-2 (+/−)×ob/ob mice, which then were bred to generate double mutants (Nscl-2 (−/−)×ob/ob). Because Nscl-2 (−/−) and ob/ob mutants are infertile it was necessary to breed double heterozygous mice to generate double mutant animals. If not indicated otherwise at least 3 male animals at the age of 6 months were used for all experiments. Isolation and analysis of adipocytes have been described before [33], in brief animals were sacrificed, and perigonadal fat pads were removed. Adipocytes were isolated by collagenase (1 mg/ml) digestion. Aliquots of adipocytes were fixed in 1% buffered osmium tetroxide and counted in a Coulter counter to determine adipocyte size distribution and cell number [37]. Determination of glucose transport, lipolysis and lipogenesis of isolated adipocytes were done immediately after isolation as described elsewhere [33]. For measurement of glucose transport isolated adipocytes were stimulated for 30 min with 80 nM insulin and incubated for 30 min with 3 µM U-^14^C-glucose. Immediately after the incubation adipocytes were fixed with osmic acid, incubated for 48 hours at 37° and the radioactivity was quantified after the cells had been decolorized. For analysis of lipolysis isolated adipocytes (100 µl of a 10% isolated fat cell suspension) were incubated in the presence of adenosine deaminase and 10 µM PIA (N6-[R-(-)-1-methyl-2-phenyl]adenosine) (basal), with 100 µM isoproterenol to produce maximal increase of lipolysis for 20 min and with 100 nM insulin, 15 min prior to the addition of 100 µM isoproterenol. Glycerol content of the incubation medium was determined after 15 min using a radiometric assay as previously described.

### Hyperinsulinemic-euglycemic clamp studies

Catheter implantation and hyperinsulinemic-euglycemic clamps of 6 male and 6 female anesthetised mice at the age of 6 months were performed as described previously [38], [39]. Experiments were started after an overnight fast. A 120-minute hyperinsulinemic-euglycemic clamp was conducted with a continuous infusion of human insulin at a rate of 20 mU/kg/min to raise plasma insulin within a physiological range. Blood samples (20 µL) were collected at 20- to 30-minute intervals for the immediate measurement of plasma glucose concentration, and 20% glucose was infused at variable rates to maintain plasma glucose at basal concentrations. Insulin-stimulated whole-body glucose flux was estimated using a prime-continuous infusion of HPLC-purified [3-^3^H]glucose (10 µCi bolus, 0.1 µCi/min; Perkin Elmer) throughout the clamps. To estimate insulin-stimulated glucose transport activity in individual tissues, 2-deoxy-D-[1-^14^C]glucose (2-[^14^C]DG; Perkin Elmer) was administered as a bolus (10 µCi) 45 minutes before the end of clamps. Blood samples (20 µL) were taken at 5, 10, 15, 25, 35 and 45 minutes after the 2-deoxy-D-glucose bolus for the determination of plasma [^3^H]glucose, and 2-[^14^C]DG concentrations. All infusions were done using microdialysis pumps (TSE Systems). At the end of clamps several tissues were isolated within a time limit of 5 minutes. Each tissue, once exposed, was dissected out within 2 seconds, frozen immediately using liquid N_2_-cooled aluminium blocks, and stored at −70°C for later analysis. For the determination of plasma [3-^3^H]glucose and 2-[^14^C]DG concentrations, plasma was deproteinized with ZnSO4 and Ba(OH)_2_, dried to remove [^3^H]H_2_O, resuspended in water, and counted in scintillation fluid (Perkin Elmer) on dual channels for separation of ^3^H and ^14^C. For the determination of tissue 2-[^14^C]DG-6-phosphate (2-DG-6-P) content, tissue samples were homogenized, and the supernatants were subjected to an ion-exchange column to separate 2-DG-6-P from 2- DG.

### Isolation of preadipocytes, flow cytometry, and *in vitro* differentiation

Perigonadal fat pads were cut into small pieces and incubated at 37°C for 40 min with 1.5 mg/ml collagenase in isolation buffer (123 mM NaCl, 5 mM KCl, 1.3 mM CaCl_2_, 5 mM Glucose, 100 mM HEPES, Pen/Strep, 4%BSA). After filtration through 100 µm nylonfilters predipocytes were collected by centrifugation and incubated in erythrocyte lysis buffer (0.154 M NH_4_Cl, 0.01 M KHCO_3_, 0.1 mM tetrasodium EDTA, pH 7.3) for 10 min at room temperature. Preadipocytes were cultured in D-MEM∶F12 (1∶1) (Gibco) with10% FCS and antibiotics. Medium was changed every second day. Differentiation was induced by adding 2 µg/ml dexamethasone, 0.5 mM IBMX and 1 µM insulin for 48–72 hours to the medium (DMEM high glucose from Gibco with 10% FCS), once the cells had reached confluency. After this induction phase the cells were cultured for 48–72 hours in DMEM with high glucose and 10% FCS with 1 µM insulin. Until day nine after induction the cells were cultured with DMEM high glucose medium with 10% FCS. Cells were fixed with 4% buffered formaldehyde and lipid containing adipocytes were identified by Oil Red O staining.

### Histology, Immunocytochemistry and Western blot and RNA analysis

Tissue embedding, sectioning, staining (Richardson-stain, Fig. 5C, Fig. 6A) and immunocytochemistry of epididymal and paired subcutaneous inguinal white adipose tissue were all performed following standard procedures [33]. Immunocytochemistry was performed employing primary rat anti-mouse MOMA-2 and rat anti-mouse F4/80 antibodies (AbD Serotec), anti-human neurofilament and CD31 (DAKO), rabbit anti-mouse peripherin (DAKO) and CD34 antibodies (Cedar Springs). As secondary antibodies HRP coupled anti-rat immunoglobulins (AbD Serotec) and Envision dual link system-HRP (Dako) were used as described previously. The specificity of all antibodies has been addressed before [13 and unpublished observations]. For quantitative analysis of MOMA-2 (macrophage and monocyte antibody-2; marker for different macrophage stages and preadipocytes) positive but F4/80 (marker for mature macrophages but not preadipocytes) negative cells, 10 adjacent MOMA-2 and F4/80 immunostained paraffin-sections of different areas within the epididymal fat pad were counted related to the number of 250 adipocytes. The difference between MOMA- 2 and F4/80 positive cells was set as the number of cells of the preadipocyte/macrophage-like lineage [40]. Western blot analysis real time quantitative RT-PCR were performed as described previously [13], [41]. For isolation of total RNA and protein from the tissue the Trizol Reagent (Invitrogen) was used. Protein extracts were separated on 12% polyacrylamid gels and blotted on nitrocellulose membrans using semidry blot technique. Membranes were blocked in TBS-T buffer supplemented with 5% nonfat dry milk for 1 hour at room temperature and incubated over night at 4 degree Celsius with anti-mouse CD 34 (Cedar Springs). Membranes were washed in TBS-T buffer before addition of secondary antibody (anti-mouse/rabbit Envision dual link system-HRP; DAKO). Specific signals were visualized using ECL Advance Western Blotting Detection Kit (GE Healthcare). For quantitative real time PCR the following specific primer pairs were used to synthesize DNA fragments from mouse fat tissue cDNA and for cloning into pGem T-Vector (Promega), which were used in dilution series as standards: Resistin: FW-5′TCCAAATGCAATAAAGAACA3′, BW-5′ GCAGAGCCACAGGAGCAG3′; Adipsin: FW-5′ GTGGCTGGTTGGGGTGTGGTCA3′, BW-5′ AAGTGTCCCTGCGGTTGCTCTC3′; C/EBP-α: FW-5′ GGGTGGAACAGCTGAGCCGTGAACT3′, BW-5′ ATCCAGCGACCCGAAACCATCCTCT3′; C/EBP-δ: FW-5′ AGCCCACTCCACCCACTT3′, BW-5′ CGACAACTCCACCAGCTTC3′; PPARγ: FW-5′ TGCGGAAGCCCTTTGGTGAC3′, BW-5′ CTTGGCGAACAGCTGAGAGGAC3′; SREBP1: FW-5′ CCACATGGCGGGAGCACAC3′, BW-5′ GCCCAGCCGGATCTACACTATG3′. 36B4 (Rplp0, ribosomal protein, large, P0), a gene which expression is unaffected by adipogenesis and inflammation, was used as normalization control.

### Analytical procedures and statistical analysis

Serum leptin was measured using the mouse Leptin RIA Kit (#ML-82K) from Linco Research. Serum insulin levels were determined by ELISA using the Rat/Mouse Insulin Kit (#EZRMI-13K, Linco Reasearch). Serum resistin and adiponectin levels were measured with a Mouse Resistin ELISA Kit (#EZMR-96K) by Linco Research and an adiponectin ELISA Kit, respectively. Glucose and insulin tolerance tests were performed as described previously [33]. All values are expressed as mean±SE if not indicated otherwise. Comparison of means between two groups was made using unpaired two-tailed Student's T-test and for more than two groups by analysis of variance (ANOVA) followed by multicomparison Tuckey test. For ANOVA analyses the F value, degrees of freedom (dF) and significance level p are given. Significance was rejected at p≥0.05 (*); p≥0.01 (**).
